# Expression and clinical significance of Glucose Regulated Proteins GRP78 (BiP) and GRP94 (GP96) in human adenocarcinomas of the esophagus

**DOI:** 10.1186/1471-2407-8-70

**Published:** 2008-03-10

**Authors:** Rupert Langer, Marcus Feith, Joerg Rüdiger Siewert, Hans-Juergen Wester, Heinz Hoefler

**Affiliations:** 1 Institute of Pathology, TU München, München, Germany; 2 Department of Surgery, TU München, München, Germany; 3 Department of Nuclear Medicine TU München, München, Germany; 4 Institutes of Pathology, HGF – National Research Center for Environment and Health München, München, Germany

## Abstract

**Background:**

Glucose regulated proteins (GRPs) are main regulators of cellular homeostasis due to their role as molecular chaperones. Moreover, the functions of GRPs suggest that they also may play important roles in cancer biology. In this study we investigated the glucose regulated proteins GRP78 (BiP) and GRP94 (GP96) in a series of human esophageal adenocarcinomas to determine their implications in cancer progression and prognosis.

**Methods:**

Formalin-fixed, paraffin-embedded tissues of primary resected esophageal (Barrett) adenocarcinomas (n = 137) and corresponding normal tissue were investigated. mRNA-gene expression levels of GRP78 and GRP94 were determined by quantitative real-time RT-PCR after mRNA extraction. Protein expression analysis was performed with immunohistochemical staining of the cases, assembled on a tissue micorarray. The results were correlated with pathologic features (pT, pN, G) and overall survival.

**Results:**

GRP78 and GRP94 mRNA were expressed in all tumors. The relative gene expression of GRP78 was significantly higher in early cancers (pT1m and pT1sm) as compared to more advanced stages (pT2 and pT3) and normal tissue (p = 0.031). Highly differentiated tumors showed also higher GRP78 mRNA levels compared to moderate and low differentiated tumors (p = 0.035). In addition, patients with higher GRP78 levels tended to show a survival benefit (p = 0.07). GRP94 mRNA-levels showed no association to pathological features or clinical outcome.

GRP78 and GRP94 protein expression was detectable by immunohistochemistry in all tumors. There was a significant correlation between a strong GRP78 protein expression and early tumor stages (pT1m and pT1sm, p = 0.038). For GRP94 low to moderate protein expression was significantly associated with earlier tumor stage (p = 0.001) and less lymph node involvement (p = 0.036). Interestingly, the patients with combined strong GRP78 and GRP94 protein expression exclusively showed either early (pT1m or pT1sm) or advanced (pT3) tumor stages and no pT2 stage (p = 0.031).

**Conclusion:**

We could demonstrate an association of GRP78 and GRP94 mRNA and protein expression with tumor stage and behaviour in esophageal adenocarcinomas. Increased expression of GRP78 may be responsible for controlling local tumor growth in early tumor stages, while high expression of GRP78 and GRP94 in advanced stages may be dependent from other factors like cellular stress reactions due to glucose deprivation, hypoxia or the hosts' immune response.

## Background

The gluose-regulated proteins (GRPs) are members of the endoplasmatic reticulum (ER) chaperone family, originally discovered as proteins inducible by glucose starvation [1]. GRP78, also referred to as immunoglobulin heavy chain binding protein (BiP) and GRP94, also referred to as GP96, are central regulators of ER function due to their roles in protein folding and controlling the activation of transmembrane ER stress sensors. GRPs control normal physiological functions under moderate levels of basal expression. Pathological conditions, such as acidosis, hypoxia or hypothermia induce their up-regulation[2].

The functions of GRPs suggest that they may also play important roles in cancer biology and in the last years knowledge about the association of GRPs and cancer has increased: studies of GRPs have been performed with cells in culture [3-7] and only a few studies were conducted with human cancers, recently for breast cancer [8,9], prostate cancer [10] or lung cancer for GRP78[11] and for lung [12], colorectal [13] and esophageal squamous cell carcinoma for GRP94 [14]. With a few exceptions there has been the general observation that higher GRP78 and GRP94 levels correlate with higher pathological grade and aggressive behaviour. However, besides intratumoral or intracellular conditions, the regulation of GRP expression in tumor cells may be dependent from exposure to various extratumoral stress factors like the potency of the immunological answer of the host, hypoxia or cytotoxic treatment[15], so that analysis of GRPs in human tumors as well should consider those potential disturbing extrinsic factors.

Adenocarcinoma of the esophagus shows a dramatically increase in incidence during the whole 20^th ^century, faster than that of any other malignancy in Western countries. Prognosis is poor with a 5-year survival generally less than 10% despite advances in diagnosis and therapy [16-20]. Consequently, many studies have investigated molecular pathogenesis of this disease as progress with this malignancy will only be made with an improved understanding of this disease.

We now performed a study to analyze the expression of GRP94 and GRP78 on mRNA and protein levels in esophageal adenocarcinomas, in order to better understand their impact in the biology of this entity and to determine their potential prognostic implications. With regard to the considerations mentioned above we aimed to analyse a homogenous collective of patients and selected primary resected tumors of 137 patients, who were not treated by prior chemo- or radiochemotherapy.

## Methods

### Patients and specimens

Paraffin-embedded tumor samples of 137 patients with primary resected esophageal adenocarcinoma from the archives of the Institute of Pathology of the Technical University of Munich were investigated. According to the classification of Siewert and Stein all tumors were AEG type I (adenocarcinoma of the distal esophagus)[21]. All patients had undergone primary surgical resection (radical transthoracic or transhiatal esophagectomy with lymphadenectomy) at the Technical University of Munich. The minimum follow-up time after surgery for surviving patients was 42 months. Overall, the mean and medium survival times were 45 months and 37 months, respectively (range 3 to 164 months). None of the patients had received neoadjuvant chemotherapy or radiochemotherapy. Out of the 16 patients with positive resection margins, 7 patients recieved adjuvant radiotherapy.

Patients' approval was secured according to local arrangements by the ethics committee (Ethikkommission der Fakultät für Medizin der Technischen Universität München, No. 1928/07). This study was performed with patients' written consent allowing molecular research to be performed on specimen obtained during surgical resection. The pT category and the pN category of the tumors were determined according to the current TNM classification[22], histological subtyping according to Lauren's classification, tumor grading according to WHO classification. Clinico-pathologic characteristics of the patients are presented in Table 1.

**Table 1:** Clinicopathologic features of 137 patients with Barrett's adenocarcinoma included in this study.

Characteristics	Data
Age	
Mean	63
Range	33–83
Gender	
Female	12 (9%)
Male	125 (91%)
pT category	
pT1	64 (47%)
	*pT1m (mucosa) 28 (20%)*
	*pT1sm (submucosa) 36 (26%)*
pT2	25 (18%)
pT3	48 (35%)
pN category	
pN0	82 (60%)
pN1	55 (40%)
Grade	
1	12 (9%)
2	61 (45%)
3	64 (46%)
Lauren's Classification	
Intestinal	119 (87%)
Mixed	15 (11%)
Diffuse	3 (2%)

### Real-Time Quantitative Reverse-Transcriptase-Polymerase Chain Reaction (RT PCR)

Microdissection, RNA extraction, cDNA synthesis and RT-PCR were performed as described previously with minor modifications[23]: From representative paraffin blocks of tumor tissue or normal squamous epithelium two 10 μm sections were deparaffinized (xylene for two times 10 min) and rehydrated (ethanol 100%/90%/70% ethanol for 5 min each). Following short hemalaun staining, a minimum of 2000 cells of defined carcinoma areas were scraped off the glass slides with a sterile blade under light microscopic control. Hemorrhagic or necrotic areas were excluded and we aimed to obtain a high percentage of tumor tissue compared to stromal components (at least 75%). The microdissected tumor tissue was transferred into a sterile 1.5-ml tube containing RNA lysis buffer. Lysis was carried out at 60°C for 24 hours until the tissue was completely solubilized.

RNA was purified by phenol and chloroform extractions followed by precipitation with an equal volume of isopropanol in the presence of 20 μl of 2 mol/L sodium acetate (pH 4.0), and 2 μl of 10 mg/ml of carrier glycogen at -20°C. The RNA pellet was washed once in 70% ethanol, dried and resuspended in 20 μl of RNase-free water.

10 μl of RNA was transcribed into cDNA by Superscript II reverse transcriptase (Invitrogen) and 250 ng of random hexamers (Roche, Penzberg, Germany) according to the manufacturer's recommendations in a final volume of 20 μl. PCR reactions were performed in at least two replicates with the Taq^®^Man Universal PCR Master Mix (15 μl, Applied Biosystems, individual Taq^®^Man Gene Expression Assays) by using 5 μl of diluted cDNA, 1 μl 200 nmol/L of the labeled probe, and 1,5 μl pre-developed primer-probe sets for GRP78 (Applied Biosystems assay ID Hs99999174_m1) and GRP94 (Applied Biosystems assay ID Hs00427665_g1) was used. Relative expression levels of target genes were determined by the relative standard curve method. Standard curves and line equations were generated by using a standard cDNA solution from SW 480 colon carcinoma cell line (fresh frozen) which was serially fivefold diluted and analyzed in duplicates for the genes of interest and GAPDH as normalizing housekeeping gene[24]. Based on the C_T _value and the corresponding standard curve, the mRNA quantity of each sample was calculated by determining the ratio between the amounts of the gene of interest and GAPDH. Primers and probe sequences for GAPDH are available from the authors on request.

### Immunohistochemical studies

#### Preparation of Tissue microarrays

From each of the 137 carcinomas one paraffin block was selected and viable, representative areas of tumor specimens were marked. Core needle biopsies were retrieved from the original tumor blocks using a manual arrayer (Beecher Instruments, Sun Prairie, WI) and positioned in a recipient paraffin array block. We aimed at obtaining at least three tissue cylinders per tumor with a diameter of 0.6 mm from each biopsy specimen.

#### Immunohistochemistry

Fresh 2 μm sections from TMA blocks were transferred to glass slides, dewaxed and rehydrated. Antigen retrieval methods were applied according to the manufacturers' recommendations. The TMA slides were cooled and incubated with the primary antibodies GRP78 (abcam, Cambridge, UK) 0.20 mg/ml diluted at 1:100 and GRP94 (Santa Cruz Biotechnology Inc, CA), 0.20 mg/ml diluted at 1:2000. The reaction was developed with the labelled streptavidin-biotin-alkaline phosphatase system with fast red used as the reaction indicator. After counterstaining with hematoxylin, slides were dehydrated in ascending concentrations of ethanol and mounted. Plasma cell staining was used as internal positive control. Staining was graded for intensity (1 = negative/weak; 2 = moderate; 3 = strong) and percentage of cells stained (1 = 0–10%; 2 = 10–50%; 3 = 50–100%) according to Lee et al[25] The overall expression index was determined based on the factor of the previous variables and classified into one of the following groups: negative/weak (1, 2), moderate (3, 4), strong (6, 9).

### Statistical analysis

SPSS statistical software was used for statistical analysis. Relative gene expression levels were expressed in quartiles. Associations in 2 × 2 tables were evaluated with Fisher's exact test. Correlations were assessed by Pearson or Spearman correlation analyses. Comparison between groups were performed using the student's T-test or by analysis of variance. Survival analysis was performed using Kaplan-Meier estimates, log rank tests and Cox's proportional hazards regression analysis. All tests were 2-sided, and the significance level was set at 5%.

## Results

### Gene Expression of GRP94 and GRP78mRNA

According to the selection criteria mentioned above mRNA analyis was performed on tissue of 70 tumors and 10 probes of normal tissue. Quantitative real time-RT-PCR analysis showed that GRP78 and GRP94 mRNA in both cancer and normal tissues were easily detectable. There was a significant, positive correlation between the relative gene expression levels of GRP78 and GRP94 (p = 0.001; r = 0.404). Table 2 shows that expression levels of GRP78 mRNA in early tumor stages were higher than in normal esophageal squamous epithelium and in tumors with advanced stages (p = 0.031), while GRP78 levels of advanced tumors were not significantly different from those in normal tissue. Furthermore, for GRP78, significant higher mRNA levels were found in the well differentiated tumors as compared to moderately and poorly differentiated tumors (p = 0.035). In contrast, tumor GRP94 mRNA levels were equal to normal esophageal squamous epithelium in all stages and failed to show any association to pathological features.

**Table 2:** Relative expression levels of GRP78 and GRP94 gene expression (mean+/-SW) and clinicopathologic features (gene/GAPDH)

Group	Cases	GRP78/GAPDH	GRP94/GAPDH
tissue			

normal	10	0,53 ± 0,45	0,47 ± 0,61
carcinoma	70	0,96 ± 0,89*	0,43 ± 0,58
			
pT-category			

pT1	41	1,15 ± 0,94^§^	0,45 ± 0,55
*pT1m*	*15*	*1,51 ± 1,08*^§§^	*0,56 ± 0,73*
*pT1sm*	*26*	*0,95 ± 0,79*	*0,39 ± 0,43*
pT2	13	0,60 ± 0,51	0,43 ± 0,83
pT3	15	0,62 ± 0,78	0,30 ± 0,35
			
pN-category			

pN0	46	1,00 ± 0,89	0,38 ± 0,53
pN1	23	0,80 ± 0,83	0,49 ± 0,67
			
differentiation			

G1	6	1,73 ± 1,00^#^	0,29 ± 0,28
G2	33	0,84 ± 0,89	0,48 ± 0,77
G3	26	0,82 ± 0,70	0,31 ± 0,27

* p = 0.022 vs. normal.

^§ ^p = 0.042 vs. pT2 and pT3.

^§§ ^p = 0.009 vs. pT2 and pT3.

^# ^p = 0.035 vs. G2 and G3

### Immunohistochemistry

**Figure 1:**
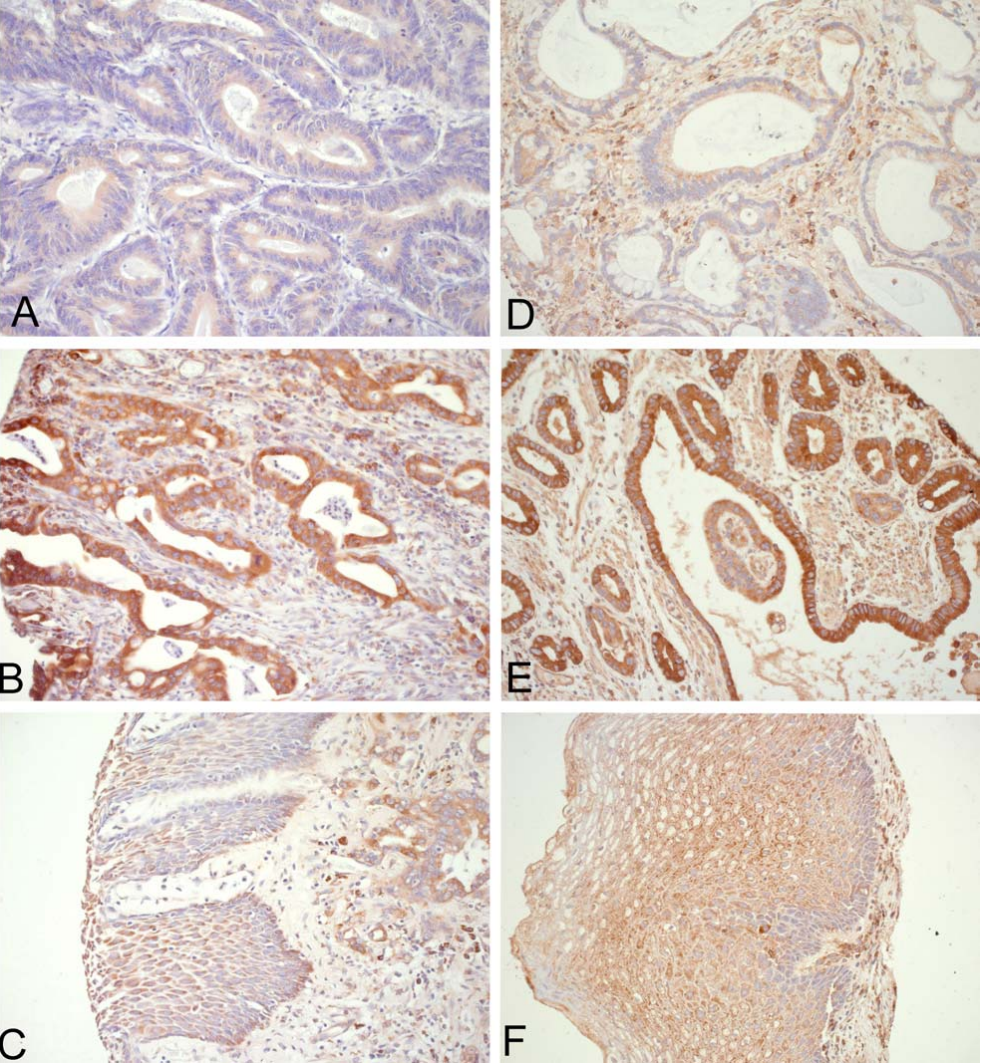
**Immunohistochemical staining for GRP78 and GRP94 in esophageal adenocarcinoma.A **Weak expression of GRP78; **B **Strong expression of GRP78; **C **moderate expression of GRP78 in normal squamous epithelium (left side) and adenocarcinoma (right side); **D **weak expression of GRP94; **E **strong expression of GRP94; **F **moderate expression of GRP94 in normal squamous epithelium. (20×).

Neoplastic tissue was interpretable in 126 cases for GRP78 and in 127 cases for GRP94. Cytoplasmatic staining was detectable in all tumors for both GRP78 and GRP94. GRP78 and GRP94 expression correlated significantly with each other (p = 0.029). Staining patterns are given in Table 3 and in Figure 1. Normal squamous epithelium showed moderate staining intensity for GRP78 and GRP94 (see also Fig 1). With regard to pathological features, there was a significant correlation between a strong GRP78 expression and early tumor stages (pT1m and pT1sm, p = 0.038). No correlation was found between GRP78 expression and lymph node involvement or tumor grade.

**Table 3:** Immunohistochemical results for GRP78 and GRP94

Group	GRP78 (n = 126)			GRP94 (n = 127)		
	negative/weak	moderate	strong	negative/weak	moderate	strong
total	49	61	16	15	61	51
						
pT-cat.						

pT1	26	27	10*	9^§^	35^§^	15
*pT1m*	*8*	*10*	*5*	*2*	*12*	*6*
*pT1sm*	*18*	*17*	*5*	*7*	*23*	*9*
pT2	13	8	1	4	10	9
pT3	10	26	4	2	16	25
						
pN-cat.						

pN0	27	37	10	8^§§^	40^§§^	22
pN1	22	24	5	7	20	27
						
differentiation						

G1	2	3	3	1	6	2
G2	17	25	6	7	25	24
G3	23	25	3	7	29	23

* p = 0.038 for pT stage and strong vs. low to moderate GRP78 expression.

^§ ^p = 0.001 for pT stage and low to moderate vs. strong GRP94 expression.

^§§ ^p = 0.036 for pN stage and low to moderate vs. strong GRP94 expression

In contrast, for GRP94 low to moderate expression was significantly associated with earlier tumor stage (p = 0.001) and less lymph node involvement (p = 0.036). Tumor grade was not associated with GRP94 expression.

### Correlation of mRNA-gene expression levels and immunohistochemistry

GRP78 mRNA expression showed a significant, positive correlation with GRP78 protein-expression evaluated by immunohistochemistry (p = 0.01). Particularly, patients with a strong GRP78 protein expression showed significant higher mRNA-levels than patients with low or moderate gene expression levels (p < 0.001, see table 4). Correlation between GRP94 mRNA gene expression levels and GRP94 protein expression was notable but not significant (p = 0.19, see table 4).

**Table 4:** Correlation between GRP78 and GRP94 gene expression and immunohistochemistry

	Cases	staining	gene-expression level (mean ± SW)
GRP78	22	negative/weak	0,75 ± 0,75
	35	moderate	0,75 ± 0,77
	9	strong	2,74 ± 1,62*

GRP94	6	negative/weak	0,12 ± 0,09
	36	moderate	0,41 ± 0,41
	18	strong	0,64 ± 0,93

* p < 0.001 vs. Negative/weak and moderate

### Combined GRP78 and GRP94 expression

Combination of GRP78 and GRP94 gene expression levels (both high GRP78/GRP94 expression levels vs. low GRP78/GRP94 levels vs. mixed GRP78/GRP94 levels; cut off: median gene expression level, see table 5.) did not show any correlation with pathological parameters. However, the patients with both strong GRP78 and GRP94 protein expression exclusively showed either early (pT1m or pT1sm) or advanced (pT3) tumor stages and no pT2 stage. Patients with combined low GRP78 and GRP94 expression had predominantly early tumor stages. Mixed GRP78/GRP94 protein-expression was heterogenous with more early tumor stages in patients with high GRP78/low GRP94 expression and more advanced tumors in patients with low GRP78/high GRP94 levels. (p = 0.031, see table 6.)

**Table 5:** Combined GRP78 and GRP94 relative gene expression levels (gene/GAPDH) and pT-category

Group	high GRP78/high GRP94	high GRP78/low GRP94	low GRP78/high GRP94	low GRP78/low GRP94
total	23	11	11	24
				
pT cat.				

pT1	18	7	6	10
pT2	3	2	1	7
pT3	2	2	4	7

p = 0.197 (X^2^-test)

**Table 6:** Immunohistochemical results for combined GRP78 and GRP94 and pT-category

Group	high GRP78/high GRP94	high GRP78/low GRP94	low GRP78/high GRP94	low GRP78/low GRP94
total	6	9	35	62
				
pT cat.				

pT1	3	7	9	34
pT2	0	1	8	12
pT3	3	1	18	16

p = 0.031 (X^2^-test)

### Survival correlations

**Figure 2:**
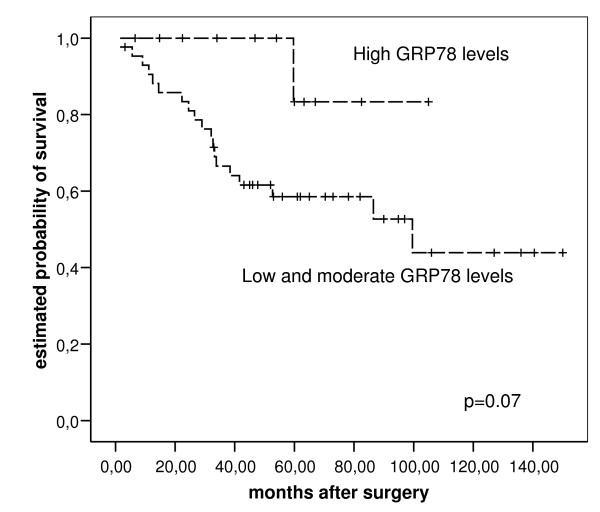
GRP78 levels in esophgeal adenocarcinoma and survival.

For overall survival analysis patients with positive resection margins, distant metastasis at the time of surgery or survival less than 1 month, were excluded. Univariate analysis using time of death as the clinical end point revealed high significant correlation of overall survival and pT-category (HR = 1.446; CI = 1.20–2.44 p < 0.001) and pN-category (HR = 2.773; CI = 1.36–3.21; p < 0.001) and a trend towards better prognosis for patients with very high GRP78 mRNA-levels (cut-off: 4^th ^quartile, HR = 1.02; CI = 0.990–1.029; p = 0.070) and for patients with high GRP94 mRNA-levels (cut-off: median, HR = 0.016; CI = 0.974–1,059; p = 0.2). However, the prognostic impact of high GPR78 and GRP94 levels was not independent from pT-and pN-category in multiple regression analysis (see fig. 2).

Immunohistochemical staining patterns of GRP78 and GRP94 protein expression failed to show any correlation with patient's survival, as well combined GRP78/GRP94 levels with survival.

## Discussion

In the present study we examined GRP78 (BiP) and GRP94 in primary resected adenocarcinomas of the esophagus on mRNA gene expression level by quantitative real time RT-PCR and on protein level by immunohistochemistry. We found (a) a positive correlation between the mRNA levels and the protein levels of GRP78 and GRP94, (b) higher GRP78 mRNA and protein expression levels in early tumor stages and tumors of higher differentiation grades, and (c) in contrast to that an association between high GRP94 protein expression and advanced tumor stage and lymph node involvement. Interestingly, in the group of patients with combined strong GRP78 and GRP94 protein expression levels, an association with either early (pT1) or advanced (pT3) tumor stages, while patients with pT2 tumors showed moderate GRP78 and GRP94 protein expression levels.

GRP78 (BiP) and GRP94 are the best studied members of the family of glucose-regulated proteins (GRPs). GRP94 and GRP78 are found constitutively within the ER and perform normal physiological functions under moderate levels of basal expression. Because of their ability to assist in protein folding and assembly, the GRPs are referred to as molecular chaperones[26]. They were first described as a set of proteins whose synthesis was enhanced when cells were deprived of glucose[1,2]. In tumor cells, besides hypoxic acidic or above mentioned glucose starvation conditions, the induction of GRP94 or GRP78 may also represent a defence mechanism for the survival of cancer cells exposed to these stress conditions or to the immunological response of the host[15,27,28].

Most studies of GRPs induction in tumors first have been conducted with animal models or cells in culture like breast cancer[4,25] or colorectal[29,30] cancer cell lines where correlated regulation of GRP78 and GRP94 gene expression was reported confirming our findings. Only one study deals with esophageal adenocarcinoma in rats and very few samples of human carcinomas[31]. In other human cancers, there has been the general observation that higher GRP78 and GRP94 levels correlate with higher pathological grade and aggressive behaviour in breast[8,9,32], liver[33], colon[13,34] and prostate[10] carcinomas. A study dealing with esophageal squamous cell carcinomas describes a higher GRP94 protein-expression in carcinomas compared to normal esophageal mucosa without further correlation to pathological characteristics [14]. In contrast, for lung cancer[11] there are conflicting reports on this association and one study demonstrates an association between high GRP78 levels and good prognosis concordant to our findings for esophageal adenocarcinomas[12]. The discrepancies between our results and others may reflect a different GRP regulation depending from the tumor type or the heterogeneity of the investigated tumor collectives.

Another interesting finding of our analysis was that high GRP78 gene and protein expression was found in early stages of esophageal adenocarcinoma and in a small proportion of patients with advanced tumor stages high GRP78 protein expression also could be detected. High GRP94 protein expression exclusively was associated with advanced tumor stages and patients with pT2 tumors showed moderate GRP78 and GRP94 levels. This observation may be explained by different mechanisms of GRP regulations: overexpression that occurs in early stages of disease may represent a reaction to an early response of the host's immune system, while upregulation in advanced stages may be related to different stress facors like glucose starvation, hypoxia or also immune reactions towards the tumor[35]. Furthermore, there are reports that show that the induction of GRP78/GRP94 at the protein level is not always corresponding with the transcript level[4]. So increased protein expression that fails to correlate with gene expression level – like in our study for GRP78 and GRP94 in advanced tumor stages- may be related to posttranslational regulations or modifications like activating or inactivating phosphorylation or glycosylation [36].

Another aspect that may draw attention to the role of GRPs in human cancers is the relationship between induction of GRPs and tumor resistance against chemotherapy (CTX) treatment, as reported very recently for breast [25] and prostate[37] cancer, where high GRP78 expression was associated with tumor resistance to CTX. However, influence of CTX on GRP expression and vice versa was not topic of our investigations. In this first study about GRPs in – to our knowledge – the largest series of human esophageal adenocarcinomas so far, we aimed to investigate GRP expression in tumors without disturbing influences of cytotoxic treatment as it strongly may influence the expression of GRPs. So we selected only patients with primary resected tumors, without prior chemo- or radiochemotherapy (RCTX) – the few patients with postoperative radiation therapy due to positive resection margins were excluded for survival analysis. Thereby we achieved knowledge about the biology of GRPs in untreated esophageal adenocarcinomas and their partly heterogenous expression patterns even in conditions without the presumably strong influence CTX/RCTX. These findings are essential informations for forthcoming studies dealing with the interaction of CTX/RCTX and GRP expression.

## Conclusion

In summary, by analysing the expression of Glucose-related proteins (GRPs) in primary resected human adenocarcinomas on mRNA gene expression level and on protein level we could demonstrate an association with tumor stage and behaviour. Our findings may help to better understand the complex mechanisms of cancer biology, immunology and tumor response to stress conditions reflected by increased GRP activation. They may serve as basis for studies that also could direct towards analyzing whether sensitivity or resistance to chemotherapeutic or other antitumoral drugs is associated with increased or decreased expression of GRPs in tumor cells and therefore help to improve individualized cancer treatment.

## Competing interests

The author(s) declare that they have no competing interests.

## Authors' contributions

RL conceived of the study, carried out assembly of tissue micro arrays and evaluation of immunohistochemical stainings and of RT-PCR data, and was involved in preparation of the manuscript. MF and JS participated in the design of the study and were responsible for collection of clinical data. HW participated in the design of the study and coordination and helped to draft the manuscript. HH conceived of the study, and participated in its design and coordination and helped to draft the manuscript. All authors read and approved the final manuscript.

## Pre-publication history

The pre-publication history for this paper can be accessed here:


